# Supervised—not voluntary—upper limb exercise enhances vestibular function in Parkinson’s disease

**DOI:** 10.3389/fneur.2025.1618719

**Published:** 2025-07-08

**Authors:** Federica Ginanneschi, David Cioncoloni, Carla Battisti, Armando Bucciarelli, Federica Dominici, Roberto Marconi, Alessandro Rossi, Lucia Monti

**Affiliations:** ^1^Department of Medical, Surgical and Neurological Sciences, AOUS, Siena, Italy; ^2^Unit of Neurology, Cardio-Thoracic-Neuro-Vascular Department, Misericordia Hospital, Grosseto, Italy

**Keywords:** Equitest, forced exercise, Parkinson, rehabilitation, vestibular system

## Abstract

**Background:**

Gait dysfunction has emerged as the greatest challenge in Parkinson disease (PD) management. Decreased vestibular efficacy may contribute to imbalance in PD. The present study aims to explore whether an upper limb aerobic exercise, performed using a device that primarily targets the axial muscles of the cervical-dorsal spine, can improve postural control and motor symptoms in PD.

**Methods:**

Twenty-eight patients with PD were evaluated before and after 2 months of exercise training, using dynamic posturography for the 6 conditions of the Sensory Integration Test (SOT) within the Equitest device, along with clinical measures. The effects of two exercise modes—supervised exercise (SE) and not supervised, voluntary exercise (VE)—were analyzed. Unified Parkinson Disease. Rating Scale (UPDRS) Part III and Hoehn and Yahr scale were used for clinical evaluation.

**Results:**

A significant improvement in vestibular SOT values was observed only in subjects belonging to the SE group (55.6 ± 14.9 pre-training vs. 65 ± 11.2 post training, *p* = 0.017). Somatosensory and visual SOT scores did not change after training in any group. Both clinical scales showed statistically significant improvement after 8 weeks of training, but only in the SE group (*p* = 0.031) for the Hoehn and Yahr scale, and *p* = 0.007 for UPDRS Part III, indicating clinical improvement in the SE group.

**Discussion:**

Active assistive SE performed with upper limbs can improve the utilization of vestibular information, and, consequently, enhance motor performance in PD patients. It should therefore be considered a crucial treatment methodology for PD patients especially those with motor limitations in the lower limbs.

## 1 Introduction

Parkinson’s disease (PD) is a progressive neurodegenerative disorder that primarily affects movement, caused by a neuronal loss of dopamine-producing neurons in the substantia nigra and resultant degeneration of dopaminergic pathways in the basal ganglia. The cardinal symptoms include tremors, muscle rigidity, bradykinesia, and balance problems. Although there is currently no cure, various treatments, including medication and physical therapy, can help manage the symptoms and improve quality of life. Postural instability is a common and disabling symptom of PD, affecting about 20% of patients at onset and rising to 90% with disease progression (1); it is thought to result from changes in static posture and postural reflexes.

Gait dysfunction has emerged as the greatest challenge in PD management, likely due to its paroxysmal nature and the complex, interconnected motor and sensory circuits underlying the different domains of postural control (2, 3). Postural control strategies rely on a complex integration of visual, vestibular and proprioceptive somatosensory inputs. Sensory processing in PD is disturbed at the subcortical and cortical levels (see Takakusakifor et al. (4) for review). An insufficient supply of cholinergic and monoaminergic input impairs sensory integration within the thalamus (5). Furthermore, the reduced cholinergic supply from the basal forebrain, determines a deficit in cortical processing of proprioception, (6, 7), vestibular graviception (8, 9), and visual sensation (10) in the parietotemporal cortex.

Recent papers have demonstrated that the inability to efficaciously utilize vestibular information to retain upright stance is a major determinant of imbalance in PD, independent from visual and somatosensory processing (11, 12). Since postural instability has been associated with disease progression and higher Unified Parkinson’s Disease Rating Scale (UPDRS) scores (13–15), therapeutic approaches aimed at enhancing the ability to utilize vestibular information could also be beneficial in addressing motor impairments in PD.

The present study aims to explore whether an upper limb aerobic exercise, performed using a device that primarily targets the axial muscles of the cervical-dorsal spine, rebalancing proprioceptive input from neck muscles (16), can improve postural control and, consequently, motor symptoms in individuals with PD. Specifically, the effects of two exercise modes—supervised exercise (SE) and not supervised, voluntary exercise (VE)—were analyzed.

Supervised exercise, is comparable to forced exercise, a form of aerobic activity that has been receiving increasing attention in the research literature, particularly in studies on PD (17–23). It involves the assistance of an external source or person to help individuals exercise at higher intensities or for longer durations than they would voluntarily consider possible. The primary distinction between the SE and VE is that the former enhances the patient’s voluntary effort to achieve a higher exercise rate, whereas VE is performed at the patient’s self-selected, typically lower, pace.

It has been suggested that contradictory results in rehabilitative treatments may be due to differences in the rate or intensity of exercise performed (17). This hypothesis indirectly highlights the distinction between VE and SE, with SE pushing patients beyond their voluntary limits.

We used the computed dynamic posturographic EquiTest® System (NeuroCom Int. Inc., Clackamas, OR, United States) (24) to assess postural stability—the ability to control the center of mass in relation to one’s base of support during both static and dynamic tasks.

## 2 Methods

### 2.1 Subjects

We enrolled 28 subjects with idiopathic PD, diagnosed according to the United Kingdom Parkinson’s Disease Society Brain Bank criteria (25). All participants had a self-reported stable and optimized daily dose of antiparkinsonian medication for at least 4 weeks prior to study enrollment (26). These subjects were recruited from the neurology outpatient clinics at Siena University Hospital and Grosseto Hospital. The subjects were required to have a score on the modified Hoehn and Yahr scale between 2.5 and 3, indicating physical independence with mild to moderate bilateral disease and some impairment of balance. During the study period, the patients did not undergo any other rehabilitative treatments or changes in their medication.

Exclusion criteria included the presence of neurological (apart from PD) and systemic diseases, visual and cognitive impairment; in addition we exclude subjects with musculoskeletal pathologies affecting the lower and/or upper limbs or the spine. Participants were informed about the study and signed a written informed consent form before joining. The Local Ethics Committee approved the study protocol (number: 14548_2019).

### 2.2 Exercise training and clinical evaluations

**Figure 1:**
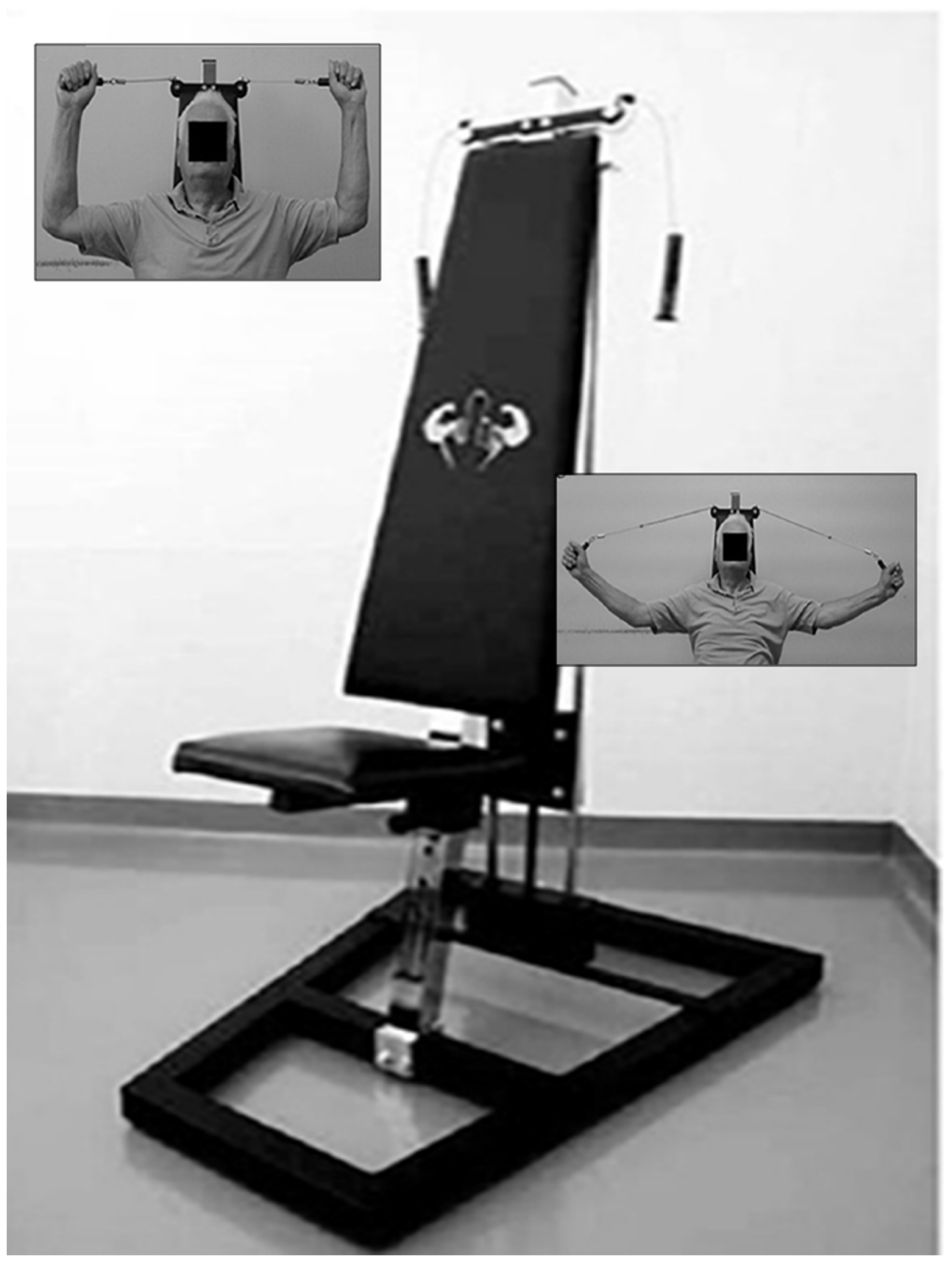
Angel’s Wings device. On the left the frontal view of the starting position of the exercise; on the right the frontal views of the distension of the forearms.

**Figure 2:**
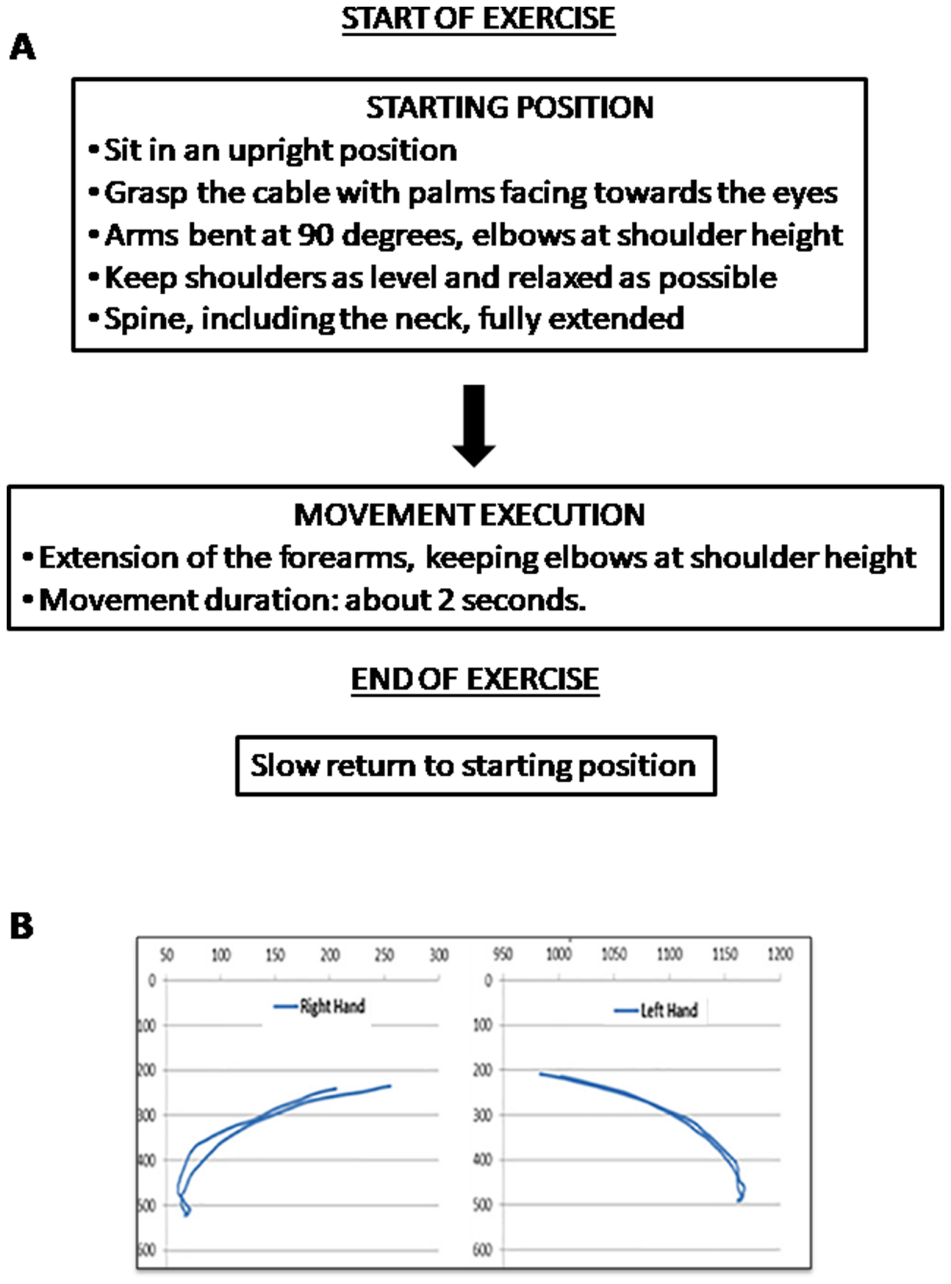
**(A)** Description of the phases that make up the exercise. **(B)** Trajectories (in pixels) of both hands required for a correct execution of the exercise with Angel’s Wings device, in an exemplificative patient.

Subjects participated in an 8-week upper-limb exercise program using Angel’s Wings (Figure 1), a patented device (patent number 0001401430). The Angel’s Wings device is designed to promote the distension of the cervical-dorsal spine while simultaneously rehabilitating the shoulder joint by repositioning it in its natural alignment, rather than in a forward-rotated position (16). This device includes a seat, adjustable based on the user’s arm length, and a system of cables and pulleys connected to adjustable weights (ranging from 1 to 7 kg), which participants lifted using two handles. The standardized exercise protocol comprised 16 sessions, conducted twice a week. The task is performed as follows: starting from a seated position, the user extends their forearms while keeping their elbows at shoulder height to lift a weight using the device’s cable system. The forearms move within the frontal plane. The weights in the device were adjusted in each series, starting with 2–3 kg in the first, increasing to 4–5 kg in the second, 6–7 kg in the third, then decreasing to 4–5 kg in the fourth, and finally returning to 2–3 kg in the fifth. This progressive increase and decrease in weight was designed to optimize muscle engagement and workout effectiveness. The exercise with the device necessitates a short learning period to be performed correctly until the correct trajectories are learned (evidenced by a reduction in errors and the development of smooth, effortless performance). In the Figure 2, the phases that make up the exercise are shown; at the bottom of the figure, the trajectories (measured in pixels) of both arms throughout the movements are presented. Trajectories and duration were monitored using the Microsoft Kinect (27).

Before the training with Angel’s Wings, all patients performed a 10-min warm-up focused primarily on upper limb movements. The warm-up did not include the Angel’s Wings exercise; rather, it consisted of joint mobilization movements, such as shoulder circles and torso twists.

The subjects were divided in two groups. In the first group (the SE group), all sessions were conducted under the supervision of a trainer who guides and encourages the patients to perform the correct exercise at a constant, pre-established rate, ensuring a full and correct completion of the exercise. In addition, the trainer could easily determine, from the quality of the movements, whether the individual patient could improve their performance. In particular, each of the five series was designed to last approximately 2 min, with a 2-min rest permitted at the end of each set. Therefore, the exercise performed in this group is conceptually similar to a forced exercise (18), which is a form of aerobic exercise performed at a higher intensity than what the subjects believed to be their maximum, requiring participants to be actively engaged in its execution.

In the second group (the VE group), once patients had learned the exercise, they performed it independently without the presence of a trainer. Specifically, they were instructed, like the participants in the other group, to perform the exercise using progressively increasing weights across the different series and at a controlled pace. However, if they felt fatigued, they were not encouraged by the trainer to complete each set for the full 2 min or to increase the device’s weight. In practice, they were free to perform the exercise at a lower intensity and slower pace if they felt tired. As a result, some patients may have been reluctant to increase the device’s weight or may have taken rest periods longer than 2 min between series.

Therefore, in the absence of external encouragement or stimulation, patients rarely exceeded their perceived maximum capacity. Although the exercise was technically performed correctly, it was generally carried out at a lower intensity and frequency. Block randomization was performed at point of enrollment, to ensure that the number of subjects in the two groups was balanced.

The clinical evaluation was performed using the original UPDRS Part-III motor examination and the Hoehn and Yahr scale (28), at time 0, before the training with Angel’s Wing, and at the end of the training period, which was approximately 7 days after the completion of the 8-week exercise protocol. All clinical evaluations were conducted by the same neurologist, who had extensive experience in Parkinson’s disease (PD) and was blinded to the participants’ group allocation.

### 2.3 Computerized dynamic posturography

Postural stability was evaluated using an instrumented platform system: the Equitest. The method was already described in detail (29). This system included a movable support surface that rotated around an axis collinear with the ankle joints, along with a movable visual surround. Strain gages within the platform measured the tangential forces and the total vertical force exerted by the feet resting on its surface. In summary, subjects stood barefoot with their ankles directly above the X-axis of the force platform and their feet equidistant from the Y-axis.

Their arms hung loosely by their sides as they faced the visual surround, maintaining optical fixation straight ahead on a small cloud drawn at the center of the scenery. While standing on the platform, subjects attempted to maintain balance in the Romberg position as sensory conditions changed. A safety harness was used to prevent falls.

To assess postural stability and balance, we employed the Sensory Organization Test (SOT), which measured the sway of the center of gravity under 6 different conditions (SOT 1–6), each comprising three trials lasting 20 s. In conditions 1 and 2, participants stood quietly with their eyes open and closed, respectively. This assessed whether sway increased when visual cues were removed and evaluated how effectively participants relied on somatosensory input. In condition 3, participants stood with their eyes open while the visual surround was sway-referenced, rendering visual cues inaccurate. In condition 4, the support surface was sway-referenced, making somatosensory cues inaccurate. Condition 5 involved standing with eyes closed on a sway-referenced support surface, assessing how participants relied on vestibular cues when visual cues were absent and somatosensory cues were unreliable. Finally, in condition 6, both the visual surround and support surface were sway-referenced, determining whether participants continued to rely on visual cues even when they were inaccurate. SOT scores for each condition range from 0 to 100, with higher scores indicating better performance, and lower scores indicating a greater risk of losing balance (24). The sensory analysis scores were computed as ratio between the mean scores of specific conditions: “Somatosensory” represents the ratio of the second to the first condition; “Visual” is the ratio of the fourth condition to the first; “Vestibular” is the ratio of the fifth to the first condition. Visual preference (PREF) highlights the reliance on visual information, calculated as the ratio of conditions with unreliable vision to those where vision is absent. A reduced PREF implies that the subject relies on visual information even when it is unreliable (24). A cumulative SOT score ranging from 0 to 100 (composite S ± OT) is also assigned to the overall test.

### 2.4 Statistical analysis

SOT data obtained before and after the 8-week exercise training program were tested for normality using the D’Agostino and Pearson normality test. To analyze the SOT values, a paired t-test was applied to assess statistical differences between pre-and post-training data, while an unpaired t-test was used to compare pre-training data between the two groups (SE and VE).

In addition, a mixed model ANOVA, using both within- (pre vs. post) and between-subjects (SE vs. VE) factors was used.

UPDRS and Hoehn and Yahr scores were presented as median with interquartile ranges (25th–75th percentiles). A Wilcoxon matched-pairs signed-rank test was used to assess statistical differences in Hoehn and Yahr and UPDRS Part III scale scores between pre-and post-training data within the SE and VE groups. Pearson’s correlation coefficient was used for the correlation analysis. Statistical significance was set at *p* < 0.05. Results are presented as mean ± SD or percentages. Cohen’s d effect sizes were calculated to assess the change from T0 to T1 in both the SE and VE groups.

## 3 Results

We recruited 28 patients with PD, 14 of whom underwent training with SE and 14 with VE. The average age of the two groups was not significantly different: 66.9 ± 7.5 and 68.6 ± 9.1, respectively.

All patients were taking L-Dopa at varying doses (range 400 mg–750 mg) and/or dopamine agonist.

The patients performed about 50–60 repetitions for each series. It is important to underline that after learning the correct trajectories, the patients’ spontaneous voluntary rate was no more than 30–40 repetitions per series; consequently, the number of repetitions performed by the VE group was generally lower than that of the SE group.

At time 0, before training with Angel’s Wing, the Hoehn and Yahr and UPDRS Part III scale scores did not differ between the two groups. For both scales, there was a statistically significant improvement at the end of the 8 weeks of training, but only in the SE group: *p* = 0.031 (Hoehn and Yahr scale), *p* = 0.007 (UPDRS Part III scale) (Table 1).

**Table 1:** Clinical and instrumental data of the 28 patients before and after training with Angel’s Wings.

Parameters	SE/VE	SE, Pre training	SE, Post training	VE, Pre training	VE, Post training	*p* value	Cohen’s d (SE/VE)
Age (mean and SD)	66.9 ± 7.5/68.6 ± 9.1					n.s.	
Sex (M-F)	9–5/8–6					n.s.	
Disease duration (mean and SD) in years	4.8 ± 1.8/4.4 ± 1.4					n.s.	
mH&Y scale (median and 25th–75th)		2.5 (2.5–3)	2.5 (2–2.5)*	2.5 (2.5–2.5)	2.5 (2.5–2.5)	*p = 0.031	−0.8/−0.5
UPDRS III (median and 25th–75th)		2 (1.75–2)	1 (1–1.25)*	2 (1.75–2)	2 (1.25–2)	*p = 0.007	−0.8/−0.3
Somatosensory SOT (mean ± SD)		97.7 ± 1.5	97.3 ± 2.3	97.2 ± 1.6	97.6 ± 3.1	n.s.	−0.2/0.1
Vestibular SOT (mean ± SD)		55.6 ± 14.9	65 ± 11.2*	46.9 ± 17.7	52.9 ± 17.2	*p = 0.017	0.8/0.4
Visual SOT (mean ± SD)		82.0 ± 8	84.8 ± 6.5	82.5 ± 5.9	86.1 ± 6.7	n.s.	0.2/0.5
Composite (mean ± SD)		76.8 ± 18.9	79.2 ± 16.7*	76.3 ± 21. 8	77.6 ± 20.3	*p = 0.0308	0.7/0.4

The patients are divided into two groups: those who underwent supervised exercise and those who performed voluntary exercise.

F, females; M, males; mH&Y, modified Hoehn and Yahr scale; n.s., not significant; SE, supervised exercise; SOT, sensory organization test; VE, voluntary exercise. The significance is calculated within each group (SE pre vs SE post and VE pre vs VE post). UPDRS score is calculated as the mean of the single scores of the 14 items (ranging from 0: normal, to 4: unable to perform or complete). The *χ*^2^ test was employed to analyze differences in sex among the two groups.

**Figure 3:**
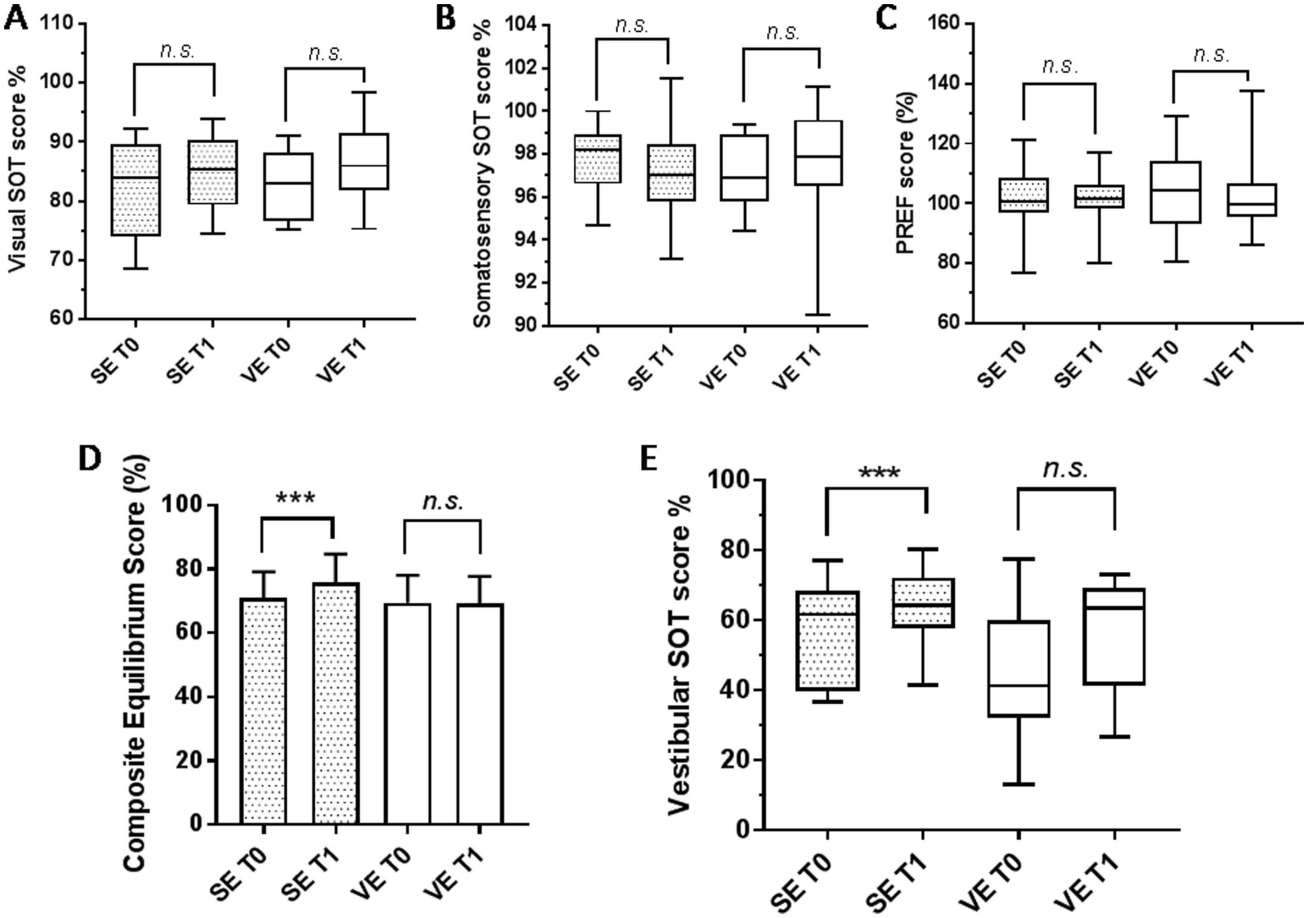
Results of the Sensory Organization Test (SOT) using the Equitest device in twenty-four patients with PD. The histograms represent the results obtained before (T0) and after (T1) training with Angel’s Wings, for Visual SOT score **(A)**, Somatosensory SOT score **(B)**, PREF (Visual preference) score **(C)**, Composite Equilibrium Score **(D)** and Vestibular SOT score **(E)**. The dotted histograms refer to the subjects who performed supervised exercise (SE), while the white histograms refer to those who performed voluntary exercise (VE). A paired t-test was applied to assess statistical differences between pre- and post-training data. A significant improvement was observed in the Composite Equilibrium Score **(D)** and Vestibular SOT score **(E)** in subjects from the SE group (*p* = 0.03 and *p* = 0.017, respectively). ***Statistically significant difference between data sets; NS, not significant.

In Figure 3 and Table 1, the results of the sensory analysis descriptive data are reported. The statistical analysis shows a significant improvement in vestibular SOT values in subjects belonging to the SE group (*p* = 0.017), with a post-training percent improvement of 17.1% (Figure 3E). Visual and somatosensory scores did not change post-training in either group. The composite subscore of the PD subjects was significantly higher post-training compared to pre-training, but only in the SE group (*p* = 0.0001) (Figure 3D).

A mixed-model ANOVA was conducted to examine the effects of Time (pre vs. post) and Group (SE vs. VE) on the vestibular SOT values. Results revealed a significant main effect of Time (*F*(1,24)=5.2, *p* = 0.031, partial *η*^2^ = 0.18), indicating that values changed from pre-to post-measurements overall. There was also a significant main effect of Group (*F*(1,24)=8.7, *p* = 0.007, partial *η*^2^ = 0.27), showing that SE and VE groups differed in their overall vestibular SOT scores. Finally, the interaction between Time and Group was significant (*F*(1,24)=4.8, *p* = 0.038, partial *η*^2^ = 0.17), suggesting that the change over time differed between the two groups.

The difference between the pre-training and post-training values of the Vestibular SOT in the SE group was analyzed for correlations with Visual SOT, Somatosensory SOT, age, and clinical scores of the subjects; however, no significant correlations were found.

No statistically significant correlation was found between UPDRS scores at baseline (time 0) and Vestibular SOT values.

## 4 Discussion

Vestibular efficacy is recognized as a key factor in abnormal postural control in PD, regardless of the extent of nigrostriatal degeneration (12). This fact may partly explain why the balance impairment in PD patients is poorly responsive to dopaminergic medications (3).

Using the SOT within the EquiTest system, we demonstrated that patients with PD and balance impairments who participated in an 8-week active SE training program exhibited improved utilization of vestibular input and showed significantly greater improvements in clinical scale scores compared to those who underwent VE training.

The difference between the pre-training and post-training values of the Vestibular SOT in the SE group did not correlate with Visual SOT, Somatosensory SOT, age, and clinical scores of the subjects. Consistent with previous research (30), the Vestibular SOT does not correlate with the initial severity of PD.

SOT creates sensory-conflict conditions, rendering the visual and/or proprioceptive balance control systems unreliable. As a result, it serves as a useful tool in rehabilitation and clinical research for assessing postural stability. The use of the SOT to study postural alterations in PD is not a novelty. Huh et al. (11) were the first to use the SOT to investigate the vestibular contributions to postural control in patients with PD, demonstrating that those with freezing of gait exhibited significantly impaired postural sensory processing, particularly an inability to utilize vestibular information. The same conclusion was reached by Bohen et al. (12), who also utilized SOT.

In our study, the improvement observed in the first group of our patients, those who underwent training through SE, cannot be attributed to any form of vestibular ‘rehabilitation,’ as no vestibular stimulation occurred under our training conditions (upper limb exercise in a seated position). There is evidence that neck proprioceptive input enhances the vestibular-evoked perception of body rotation (31). This is supported by findings that the cerebellar interpositus nucleus integrates both vestibular and neck proprioceptive signal (32). In this regard, exercise with the Angel’s Wings device, by promoting relaxation of the upper trapezius muscle and correcting the posture of the cervico-dorsal spine (16), could help rebalance proprioceptive input from the neck muscles, thereby contributing to the improvement of the vestibular function. However, the lack of improvement in patients who performed the training with Angel’s Wings voluntarily, without a trainer and likely not at their full potential, suggests that the anatomo/physiological changes above described, may not be entirely responsible for the clinical/vestibular improvement.

The most plausible hypothesis is that, similarly to what has been demonstrated in other studies on patients with PD, it is the SE itself that led to the improvement (17, 18).

When the subjects performed an exercise with a trainer (that is a SE), the exercise rate is mechanically increased to help participants achieve and maintain a pace greater than their preferred voluntary exercise rate, requiring them to be actively engaged. The exercise we used corresponds to a forced exercise as the exercise was performed at a rate higher than what our patients would spontaneously consider their maximum, facilitated by continuous guidance from a trainer.

Our result regarding the improvement in the ability to utilize vestibular information after exercise does not represent the true novelty of this work, as this data has already been demonstrated in other studies (11, 12); rather, the novelty lies in the fact that the result was achieved through a SE performed with the upper limbs. In fact, almost all the data in the literature refers to forced exercise involving the lower limbs, particularly those performed with a modified bicycle.

Forced exercise may alter cortical excitability in patients with PD by increasing both the quantity (faster motor activity) and consistency (lower variability) of afferent information compared to VE (17). It has been proposed the forced exercise determines causes hyperstimulation of peripheral sensory receptors (such as joint receptors, muscle spindles, and Golgi tendon organs), enhancing the afferent sensory input to cortical and subcortical regions, leading to increased cortical activity and motor output (18, 19, 22). Indeed, a previous study utilizing the same upper limb exercise training demonstrated changes in the recruitment efficiency of the corticomotor pathway, accompanied by an improvement in the UPDRS-III score (33).

With respect to VE, engaging subjects in SE leads to the activation of a greater number of peripheral sensory receptors, primarily the Golgi tendon organs. This helps enhance sensory afferent neural activity, which in turn stimulates central processing of information to improve motor control and performance (19, 21).

It is important to note in this context that, in patients with PD undergoing forced exercise, neuroimaging studies have confirmed that this exercise can trigger adaptive/neuroplastic brain changes (23, 34). Cerebral blood flow analysis and functional connectivity studies have shown widespread post-exercise changes in activation patterns compared to baseline, involving the supplementary motor area, primary motor cortex, basal ganglia, thalamus, and cerebellum (23). The similarity in the pattern and extent of brain activation observed on fMRI between forced exercise and antiparkinsonian drugs suggests a potential shared underlying mechanism of action in the brain that contributes to symptomatic relief. Lastly, based on findings from animal studies, forced exercise may facilitate the release of neurotrophic factors such as GDNF or BDNF, which are thought to contribute to improved motor function (35). The lack of clinical improvement in the group of patients who underwent voluntary exercise (VE), despite achieving postural correction, might be explained by the absence of the aforementioned neuroplastic/neurotrophic changes.

A limitation of our study is the relatively small sample size; consequently, we cannot completely rule out selection bias, which may limit the generalizability of our findings.

Another limitation is the lack of long-term follow-up, which prevents us from assessing the duration of the observed effects. Finally, we used the original UPDRS scale; therefore, this should be taken into account when comparing our results with studies employing the MDS-UPDRS scale.

In conclusion, we have demonstrated that SE performed with the upper limbs while seated can improve the utilization of vestibular information and, consequently, could enhance motor performance in patients with PD. The same results were not achieved through the same exercise performed voluntarily. Active assistive SE performed with upper limbs, should therefore be considered a crucial treatment methodology for patients with PD, especially those with motor limitations in the lower limbs.

## Data Availability

The raw data supporting the conclusions of this article will be made available by the authors, without undue reservation.
